# Lung cancer associated with cystic airspaces: current status and challenges

**DOI:** 10.3389/fonc.2025.1634835

**Published:** 2025-08-25

**Authors:** Linlin Wang, Yong Feng, Shiyuan Song, Jiandong Cao, Dongxu Zhang, Hong Yang, Yi Ren

**Affiliations:** ^1^ Department of Thoracic Surgery, Shenyang Tenth People’s Hospital, Shenyang, Liaoning, China; ^2^ Department of Radiology, Shenyang Tenth People’s Hospital, Shenyang, Liaoning, China; ^3^ Department of Pathology, Shenyang Tenth People’s Hospital, Shenyang, Liaoning, China

**Keywords:** lung cancer associated with cystic airspaces (LCCA), imaging phenotype, diagnosis, pathology, challenges

## Abstract

Lung cancer associated with cystic airspaces (LCCA) refers to primary lung cancers presenting with cystic airspaces accompanied by solid components, representing a relatively uncommon imaging and pathological phenotype. Although high-resolution imaging techniques, such as computerized tomography, are the primary diagnostic tools, early diagnosis remains challenging due to the similarity of its symptoms to other pulmonary diseases. Treatment options include surgery, chemotherapy, radiotherapy, targeted therapy, and immunotherapy. However, due to the unique nature of LCCA, treatment requires a highly individualized approach. The main challenges currently faced include improving diagnostic accuracy, determining the optimal treatment strategies, and advancing the understanding of its underlying biological characteristics. Future research will focus on optimizing diagnostic techniques, developing targeted therapies for specific molecular markers, exploring the application of immunotherapies, and promoting multidisciplinary collaboration. With continuous advancements in technology and personalized treatment strategies, significant improvements in the diagnosis and treatment of LCCA are anticipated. This review summarizes the epidemiology, imaging features, pathological characteristics, pathogenesis, treatment strategies, and challenges of LCCA, and offers perspectives on future research directions.

## 1 Introduction

Lung cancer associated with cystic airspaces (LCCA) represents a relatively uncommon and challenging imaging phenotype of lung cancer, distinguished by cystic lesions and complex pathological features, which have drawn significant clinical attention (1). Despite the high overall incidence and mortality of lung cancer, LCCA accounts for only a small fraction of all lung cancer cases, limiting both the recognition and research into this subtype. The hallmark of LCCA is its characteristic cystic structure, which typically presents as cystic lesions on imaging, with cysts that may exhibit either thick or thin walls and may contain fluid or necrotic material (2). These nonspecific imaging features not only complicate the diagnostic process but also increase the risk of misdiagnosis, as LCCA can be easily confused with other benign cystic pulmonary conditions such as lung abscesses, pulmonary cysts, or cystic bronchiectasis, leading to potential misdiagnoses and inappropriate treatments (3).

Moreover, the epidemiological data on LCCA remain scarce, hindering a comprehensive understanding of its etiology and pathogenesis. Existing studies are primarily confined to case reports or small-scale retrospective analyses (4, 5), making it difficult to fully characterize the clinical features of LCCA. Due to the diversity in its pathological features and the nonspecific nature of its imaging findings, both the diagnosis and treatment of LCCA present significant challenges. Furthermore, the prognosis of LCCA differs notably from that of conventional non-small cell lung cancer (NSCLC), adding to the complexity of treatment management.

Given the diagnostic challenges and current limitations in treatment options, this review aims to provide a comprehensive narrative overview of the existing literature on LCCA, summarizing epidemiology, imaging, pathology, treatment, and challenges. Additionally, we will explore the future research directions in LCCA, offering valuable insights that could improve clinical management and treatment outcomes.

## 2 Definition and classification of LCCA

LCCA represents a unique imaging and pathological manifestation of lung cancer characterized primarily by cystic lesions within the lung parenchyma. It is not recognized as a separate histological subtype in the current WHO classification but rather as a distinctive radiological and pathological phenotype seen across various NSCLC types. These lesions typically present as thin- or thick-walled cysts, which may contain fluid, necrotic tissue, or both (6). The presence of cystic structures distinguishes LCCA morphologically from the more common solid types of lung cancer. Due to its rarity and the diversity of its pathological features, LCCA poses a complex classification challenge. It is generally considered a subtype of NSCLC, but can exhibit various histological forms, including adenocarcinoma, squamous cell carcinoma, and poorly differentiated carcinoma, with pulmonary adenocarcinoma being the most common histological subtype (7, 8). The cystic changes associated with different histological types can influence clinical presentation and prognosis, thereby impacting the choice of treatment.

From a biological perspective, LCCA can be classified into low-grade and high-grade malignancies. Low-grade tumors typically grow slowly with more stable cystic structures, while high-grade tumors exhibit rapid growth with significant changes in the cystic structures, leading to a poorer prognosis. Additionally, LCCA can be categorized based on the underlying cause of the cystic formation, into necrotic, mucinous, and inflammatory types. Necrotic LCCA usually originates from tumor necrosis at the center, resulting in cavities filled with fluid or necrotic tissue; mucinous LCCA is characterized by the secretion of abundant mucin by the tumor, leading to cystic changes, and is commonly seen in adenocarcinoma; inflammatory LCCA is associated with severe inflammatory reactions caused by the tumor, resulting in tissue destruction and the formation of cystic structures.

From an imaging perspective, LCCA can further be divided into isolated, multiple, and complex cystic lesions (9). Isolated cystic lesions typically present as a single cystic structure and are more commonly seen in early or localized LCCA. Multiple cystic lesions are characterized by multiple cysts, which may indicate multifocal disease or tumor progression. Complex cystic lesions typically exhibit irregular or thickened cyst walls, and may have internal septations, suggesting malignant features.

## 3 Epidemiology of LCCA

LCCA is a rare subtype of lung cancer, accounting for approximately 1% to 4% of all NSCLC cases (10). Due to its low incidence and the similarity of its imaging features to other cystic lung diseases, such as alveolar epithelial lesions, early diagnosis can be challenging. LCCA typically affects individuals aged 40 to 70 years. Studies by Farooqi et al. (11) suggest a balanced gender ratio, while Fintelmann et al. (12) report a higher incidence in women compared to men. Smoking is a well-established risk factor for LCCA, with the majority of cases occurring in current or former smokers (6). Environmental factors, such as air pollution and occupational exposures, along with genetic susceptibility, may contribute to its pathogenesis. However, research in this area is still in its early stages. The molecular characteristics of LCCA may influence its pathogenesis to some extent, but the results of various studies have not yet reached a consensus. Given the rarity of LCCA and the lack of large-scale epidemiological data, relevant studies remain limited, highlighting the need for further exploration of its molecular mechanisms and epidemiological features.

## 4 Imaging characteristics of LCCA

LCCA typically presents on CT scans as a coexistence of mass and cystic spaces. The cystic cavities appear as low-density areas, with varying sizes, usually round or oval in shape, and well-defined borders. The cyst walls may exhibit irregular thickening. The mass component can be solid, partially solid, or ground-glass change, with irregular or spiculated borders often indicating the malignant nature of the tumor (13, 14). In some cases, patients with LCCA may show a fluid-air level, particularly when the tumor undergoes rupture or hemorrhage, resulting in a visible fluid-air interface on CT imaging. Additionally, the cyst wall may show uneven thickening, often associated with infiltrative tumor growth, further suggesting malignancy. LCCA may also invade adjacent airways and blood vessels, resulting in airway narrowing, obstruction, or vascular compression, indicating local tumor infiltration. Enlarged mediastinal or hilar lymph nodes are common imaging findings, suggesting local metastasis or invasion. As the disease progresses, distant metastasis may occur, presenting as small nodules or masses outside the lungs. Contrast-enhanced CT plays a significant role in evaluating LCCA, as the cystic component typically shows little enhancement, whereas the solid component enhances significantly, indicating the tumor’s vascularity.

**Figure 1:**
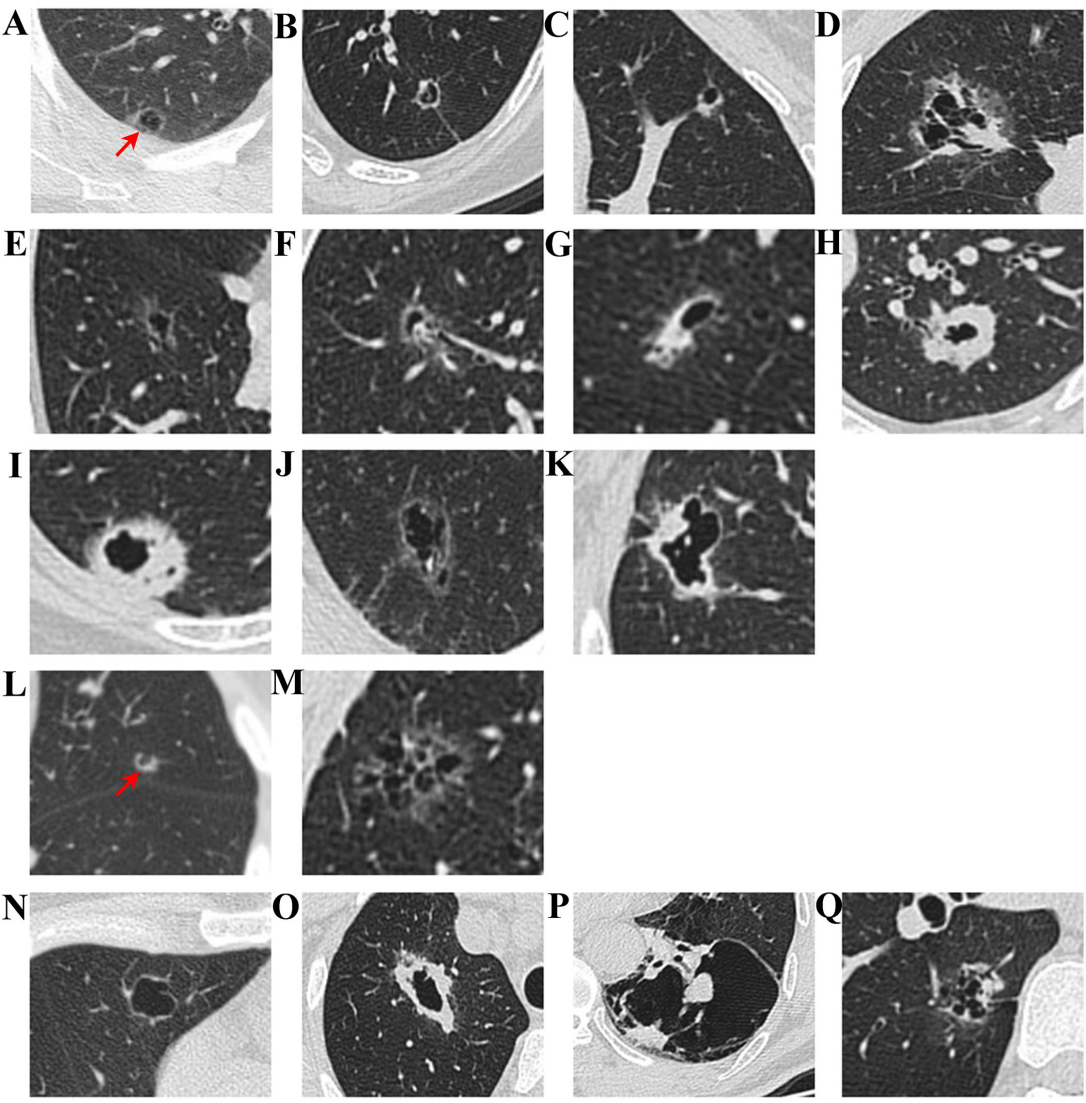
CT imaging classification of LCCA. According to Mascalchi et al., LCCA can be classified into four distinct types: Type I **(A)** refers to nodules or masses protruding from the cyst wall; Type II **(B)** denotes nodules or masses within the cyst; Type III **(C)** is characterized by soft tissue extending along the cyst wall; and Type IV **(D)** involves soft tissue distributed within the cyst. Building upon the findings of Fintelmann et al., LCCA can be further stratified based on three key parameters: the characteristics of cystic airspaces, the composition of nodules or wall thickening, and the structural configuration of cystic airspaces. With respect to cystic airspace characteristics, LCCA is classified into thin-walled tumors **(E)**, tumors with endophytic nodules **(F)**, tumors with exophytic nodules **(G)**, and thick-walled tumors **(H)**. In terms of the composition of nodules or wall thickening, LCCA is categorized into solid component tumors **(I)**, non-solid component tumors **(J)**, and partially solid component tumors **(K)**. Regarding the structural architecture of cystic airspaces, LCCA is further divided into unilocular tumors **(L)** and multilocular tumors **(M)**. Additionally, as reported by Shen et al., LCCA can be divided into four types: Type I **(N)**: Thin-walled type, with a wall thickness of less than 2 mm, Type II **(O)**: Thick-walled type, with a wall thickness greater than or equal to 2 mm, Type III **(P)**: Cystic airspace with wall nodules, and Type IV **(Q)**: Mixed type, characterized by multiple cystic airspace clusters with mixed solid or ground-glass opacities.

Regarding CT imaging grading, Mascalchi et al. (15) classified LCCA based on the size, shape, and wall characteristics of the cystic structure, finding that LCCA often presents with cystic cavities of varying sizes and thick, irregular walls, frequently accompanied by enhancement rings and solid masses (**Figures 1A-D**). Fintelmann et al. (12) emphasized the importance of cyst size, structural integrity, and the contents of the cyst (such as gas, fluid, or solid masses) in grading, while also exploring how to distinguish LCCA from other pulmonary lesions (**Figures 1E-M**). Shen et al. (16) suggested grading based on cyst size, wall enhancement, and lung tissue infiltration, with irregular borders and possibly enhanced wall layers and solid components (**Figures 1N-Q**). Jung et al. (17) noted that the imaging features of LCCA sometimes resemble benign pulmonary lesions, with single or multiple cysts, and some cases showing irregular enhancement within the cyst. The thickness and structure of the cyst wall are important factors in grading (**Table 1**).

**Table 1:** CT imaging grading criteria and descriptions of LCCA.

Researcher	Classification	Description
Mascalchi et al. (15)	Type I	Small nodules protruding from the cystic wall.
	Type II	Small nodules confined within the cystic cavity.
	Type III	Soft tissue densities extending along the cyst wall.
	Type IV	Mixed solid or non-solid tissue within multiple cystic cavities.
Fintelmann et al. (12)	(1) Cystic lesion types	a: Thin-walled; b: Endophytic nodules; c: Exophytic nodules; d: Thick-walled.
	(2) Nature of wall nodules or thickening	a: Solid; b: Non-solid; c: Partially solid.
	(3) Number of cystic spaces	a: Unilocular; b: Multilocular.
Shen et al. (16)	Type I	Thin-walled, with wall thickness < 2 mm.
	Type II	Thick-walled, with wall thickness ≥ 2 mm.
	Type III	Nodules within the wall, featuring solid nodules.
	Type IV	Mixed type, with solid or ground-glass components in multiple cysts.
Jung et al. (17)	Stage 1	Cystic airspaces appear at the center of non-solid nodules.
	Stage 2	Enlargement of the cystic airspaces.
	Stage 3	Solid components appear at the cystic borders.
	Stage 4	Solid components surround and thicken the cyst wall, causing cystic contraction.

PET-CT plays a crucial role in the diagnosis and management of lung tumors. Using the 18F-FDG tracer, PET-CT can assess the metabolic activity of the tumor, aiding in the differentiation of malignant from benign lesions. It is also useful in detecting lymph node metastasis, distant metastasis, and local tumor extension, especially for small lesions that are not visible on CT. Preoperatively, PET-CT helps with tumor staging and assessing the extent of invasion; during treatment, it monitors efficacy and can detect recurrence or metastasis early. However, due to individual variability in the uptake of fluorodeoxyglucose, which may be absent in some cases (18), the application of PET-CT in LCCA remains somewhat limited. More long-term studies are needed to further clarify its diagnostic value.

Dynamic contrast-enhanced magnetic resonance imaging (DCE-MRI) (19, 20) is primarily used to evaluate the hemodynamic characteristics and microvascular structure of tumors in LCCA. By observing the enhancement pattern of the contrast agent, DCE-MRI can help differentiate malignant from benign lesions, as malignant tumors typically show higher angiogenesis and rapid enhancement (21). DCE-MRI can also assess the permeability of tumor microvessels, which is particularly valuable in detecting solid nodules within LCCA. Karaman et al. (22) found that DCE-MRI has diagnostic value for uncertain cystic pulmonary lesions, particularly in evaluating irregular tumor wall features, with greater sensitivity than CT. However, the overall reliability and positive predictive value of DCE-MRI in diagnosing LCCA specifically remain to be established in larger clinical studies. At present, DCE-MRI should be considered as a useful adjunct tool in cases where CT findings are inconclusive, especially for evaluating irregular cyst walls or identifying small solid components within cystic lesions. Due to the limited evidence and lack of standardized diagnostic criteria, DCE-MRI is not currently recommended as a routine diagnostic modality for LCCA, and further validation in prospective studies is warranted.

## 5 Pathological features of LCCA

The pathological features of LCCA are characterized by the coexistence of cystic and solid components. The cystic areas typically consist of large, irregular cavities or cysts filled with fluid or mucinous material, with thin walls that appear as low-density on imaging (23). The cysts may be unilocular or multilocular, and some cystic walls may exhibit spiculated or undulating features. The cystic areas often result from tumor necrosis, hemorrhage, or cavitation, while the solid component consists of irregularly proliferating adenocarcinoma cells, which vary in size, with some cells secreting mucin. The boundary between the cystic and solid components is often indistinct, and the tumor exhibits significant atypia and proliferative activity. The most common histological subtype is adenocarcinoma (24), with many cases showing glandular differentiation and the presence of secretions. Histopathological examination typically reveals glands or acinar structures with minimal atypia, often accompanied by secretions. Immunohistochemical staining commonly shows positive results for adenocarcinoma markers such as thyroid transcription factor-1 (TTF-1) and Napsin A, with some cases also showing positivity for CEA and CK7.

**Figure 2:**
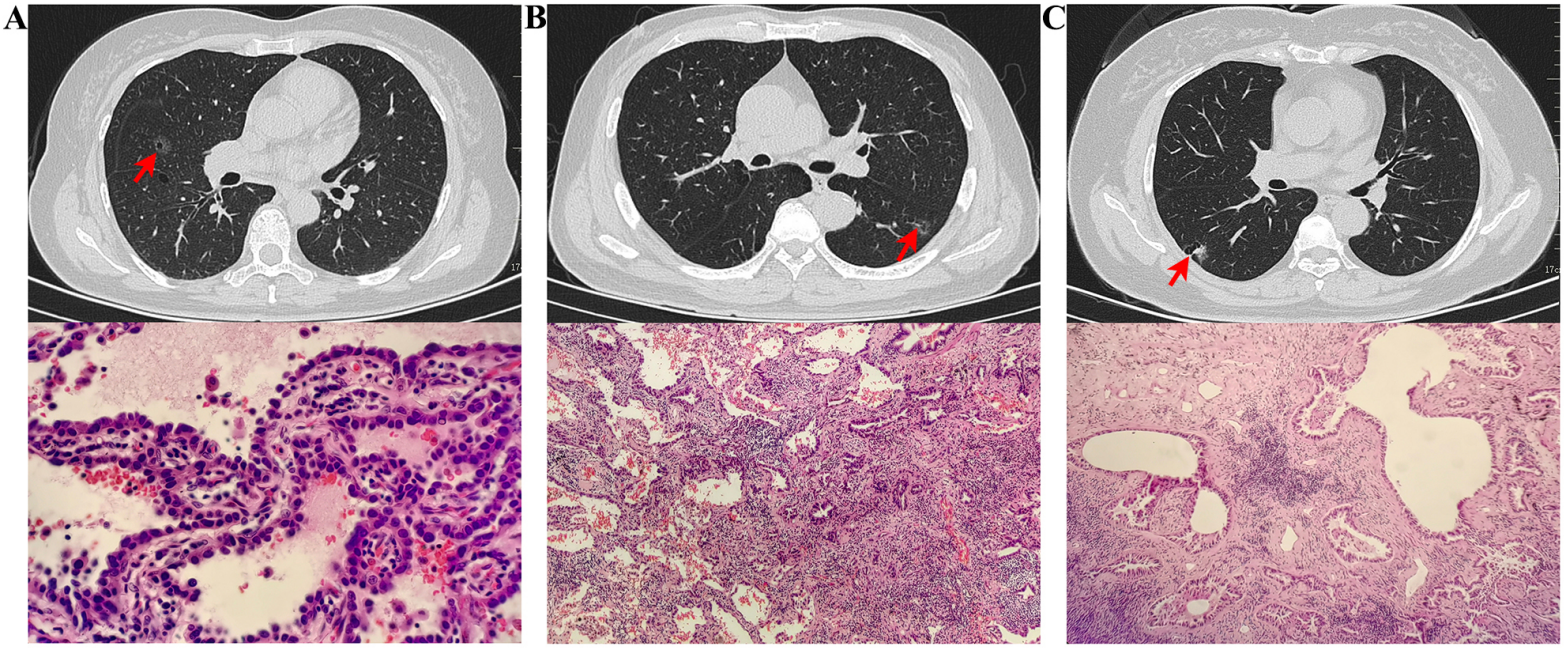
Radiologic and pathologic features of LCCA: a comparison of adenocarcinoma in situ **(A)**, Minimally Invasive Adenocarcinoma **(B)**, and Invasive Adenocarcinoma **(C)**. **(A)** CT scan revealed a ground-glass nodule in the middle lobe of the right lung, with a small lucent area inside. Pathological findings showed cancer cells growing in a single layer along the alveolar walls, with dense cell arrangement but no vascular or lymphatic invasion. **(B)** CT scan revealed a ground-glass nodule in the lower lobe of the left lung, with visible vacuoles and lobulation. Pathological findings showed most cancer cells growing along the alveolar walls, with focal stromal fibrosis and visible acinar structures, measuring less than 5 mm in size. **(C)** CT scan revealed a cavitary nodule in the lower lobe of the right lung, with pleural traction nearby and traversing blood vessels inside. Pathological findings indicated invasive growth of cancer tissue with acinar structures.

The imaging and pathological features differ between *in situ* adenocarcinoma, minimally invasive adenocarcinoma, and invasive adenocarcinoma (**Figure 2**). Adenocarcinoma *in situ* appears as a ground-glass nodule with cancer cells growing along the alveolar walls and no invasion. Minimally invasive adenocarcinoma shows a ground-glass nodule with vacuoles and lobulation, and tumor size under 5 mm. Invasive adenocarcinoma is a cavitary nodule with pleural traction and vascular invasion, with cancer cells showing invasive growth. In addition, there are distinct differences in the radiological and pathological features between benign pulmonary tumors (such as bronchiectasis with chronic inflammation, pulmonary granulomas, and pulmonary cysts with aspergillosis) and malignant tumors (such as squamous cell carcinoma, large cell carcinoma, and mucinous adenocarcinoma). Benign lesions typically present as well-defined cystic structures, with pathology showing cellular proliferation, chronic inflammatory infiltration, and necrosis. In contrast, malignant tumors are characterized by irregular or ill-defined lesions, accompanied by tumor cell infiltration, vascular invasion, and other malignant features, with pathology often revealing atypia, increased proliferative activity, and secretion. A comprehensive evaluation of both radiological and pathological findings provides essential support for clinical diagnosis and differentiation of these pulmonary diseases.

The diagnosis of LCCA requires a combination of imaging, pathological, and molecular features to ensure accurate identification and the formulation of an individualized treatment plan. Studies have shown that some patients with LCCA may harbor molecular mutations, including EGFR, KRAS, and ALK gene rearrangements, which are more commonly found in NSCLC, particularly in lung adenocarcinoma (16, 25).

## 6 Pathogenesis of LCCA

LCCA constitutes a rare and intricate radiological-pathological phenotype of lung cancer, defined by prominent cystic morphology and most commonly encountered in lung adenocarcinoma. Its pathogenesis involves multiple factors. First, abnormal tumor cell proliferation leads to space occupation in the lung, and due to poor differentiation, regional necrosis and fluid accumulation occur, resulting in the formation of cavities. Rapid tumor proliferation frequently causes incomplete or immature vascular development, leading to insufficient blood supply to the tumor’s central region, which in turn triggers hypoxia and cell necrosis. As the tumor grows, the necrotic areas gradually expand, and the necrotic tissue liquefies, forming cavities or cystic spaces (26). Additionally, secretory glands, mucinous glands, and other structures within the tumor may secrete large amounts of fluid, further expanding the cavity. The tumor may also develop a “check-valve mechanism” (27–29), which allows fluids and gases to enter but prevents their expulsion, resulting in fluid accumulation and gradual enlargement of the cavity, altering its shape. Furthermore, the tumor may cause partial airway obstruction, leading to impaired ventilation of the alveoli or bronchi. Gas accumulation may form a gas-liquid level, and the tumor may damage the alveolar walls, further promoting cystic changes. In addition, tumor cells may proliferate within pre-existing cystic spaces or invade the surrounding lung tissue, contributing to the heterogeneous and complex morphology observed in LCCA. This unique growth pattern not only increases the diversity of LCCA presentations but also adds to the diagnostic and therapeutic challenges. Factors in the tumor microenvironment, such as angiogenesis, cytokine release, and the involvement of immune cells (30), also influence tumor growth and transformation. As the microenvironment changes, some areas may become hypoxic, further inducing necrosis and cystic degeneration. Furthermore, cystic air spaces may arise from the destruction and dilation of the bronchus due to abnormal proliferation and/or the growth and infiltration of tumor cells (27). At the genetic level, lung adenocarcinoma is often associated with mutations in EGFR, KRAS, and TP53 genes. EGFR mutations promote tumor cell proliferation, survival, and migration by activating downstream signaling pathways such as PI3K/AKT and MAPK, and may also lead to abnormal angiogenesis and hypoxia. KRAS mutations, through the activation of the Ras-MAPK signaling pathway, enhance cell proliferation, apoptosis resistance, and alter the tumor microenvironment, further facilitating liquefactive necrosis and cystic change formation. TP53 mutations may lead to uncontrolled cell cycle progression, exacerbating tumor proliferation and the process of necrotic liquefaction.

## 7 Treatment strategies for LCCA

For early-stage, localized LCCA, surgical resection–typically via lobectomy or segmentectomy–remains the most common and effective therapeutic strategy. However, the distinct biological features and imaging characteristics of LCCA often limit the accuracy of the traditional TNM staging system, complicating clinical decision-making. The presence of cystic airspaces increases the risk of intraoperative cyst rupture and tumor cell dissemination, which may negatively impact prognosis. Furthermore, the cystic lesions in LCCA are frequently irregular and multifocal, with indistinct borders between tumor and normal lung tissue. These features make it challenging to determine the optimal extent of resection and lymph node dissection, necessitating more individualized surgical planning and intraoperative management.

Advancements in minimally invasive techniques, such as video-assisted thoracoscopic surgery (VATS) and robot-assisted thoracoscopic surgery (RATS), have led to their increasing adoption in NSCLC, owing to their reduced invasiveness, faster postoperative recovery, and decreased pain (31, 32). Nevertheless, evidence supporting the safety and efficacy of these approaches specifically in LCCA remains limited, and further studies are warranted. In a study by Ma et al. (8), the five-year overall survival (OS) for cystic adenocarcinomas was significantly higher than for cystic squamous cell carcinomas (66.9% vs. 48.2%, P < 0.001), with type II cystic lung cancer demonstrating the poorest prognosis. Further research is required to clarify the risks, benefits, and optimal management of fragile cystic lesions in the context of minimally invasive surgery for LCCA, with the aim of minimizing tumor dissemination.

For patients with advanced LCCA who are not surgical candidates, adjuvant therapies including chemotherapy, targeted therapy, and immunotherapy may be considered. The choice of treatment is generally guided by pathological characteristics and molecular biomarkers. LCCA is often associated with gene mutations such as KRAS and EGFR, which may influence therapeutic response and prognosis (8, 12). However, the molecular mechanisms underlying LCCA remain incompletely understood, and clinical evidence supporting targeted therapies in this population is currently lacking. Importantly, programmed death-ligand 1 (PD-L1), a key biomarker for immune checkpoint inhibitor therapy, has shown promising efficacy in NSCLC, though its specific relevance in LCCA warrants further investigation. Epigenetic regulation in LCCA is also an emerging field; ongoing advances in molecular biology have prompted investigations into the roles of DNA methylation, histone modification, and non-coding RNAs in LCCA pathogenesis. These epigenetic changes may ultimately provide novel therapeutic targets for this challenging subtype.

## 8 Current challenges and future research directions

The management of LCCA remains highly challenging, primarily due to the absence of disease-specific, standardized clinical guidelines. Most current therapeutic recommendations are still extrapolated from general NSCLC protocols, which may not adequately address the unique clinical needs of patients with cystic tumors. Evidence supporting the efficacy of adjuvant therapies–including chemotherapy, targeted therapy, and immunotherapy–in LCCA remains limited, and their precise roles have yet to be clarified. The distinctive biological and molecular features of LCCA, such as its mutational landscape and tumor microenvironment, may significantly influence therapeutic response; however, robust clinical data are still lacking. These gaps underscore the urgent need for individualized treatment strategies, enhanced multidisciplinary collaboration, and the development of dedicated clinical guidelines tailored to LCCA. In addition, early diagnosis of LCCA remains problematic. Its imaging characteristics often mimic those of benign pulmonary diseases, and the lack of specific biomarkers markedly increases the risk of misdiagnosis or missed diagnosis. These limitations not only hinder accurate prognostic assessment, but also pose significant challenges for screening, therapeutic monitoring, and long-term patient management.

Given the multifaceted challenges in the field of LCCA, future research should prioritize the following directions: establishing multicenter, high-quality registries and biobanks to provide a solid foundation for systematic epidemiological and translational studies; advancing AI-powered imaging technologies for early detection and precise disease classification; conducting prospective studies to systematically identify and validate LCCA-specific biomarkers for early diagnosis and real-time therapeutic monitoring; leveraging integrative multi-omics and single-cell approaches to unravel the molecular landscape and immune microenvironment of LCCA; developing individualized therapeutic strategies and prospective clinical trials guided by molecular and immunological profiling to accelerate precision medicine; formulating and continuously updating evidence-based guidelines for the diagnosis, classification, and management of LCCA; and undertaking long-term cohort studies to systematically identify key prognostic factors, ultimately optimizing patient outcomes.

## 9 Conclusion

Compared with other types of non-small cell lung cancer, LCCA, as a distinct radiological and pathological phenotype, poses unique and complex challenges in both diagnosis and treatment. Its characteristic manifestations, together with the absence of standardized clinical guidelines and a lack of robust therapeutic evidence, underscore the urgent need for more systematic research focused on LCCA. Moving forward, elucidating the molecular underpinnings of LCCA, advancing early and precise diagnostic approaches, and developing individualized therapeutic strategies will be essential for improving clinical outcomes in this patient population.
